# Supplementary material for: Bicyclomycin generates ROS and blocks cell division in Escherichia coli
Source: PLoS One. 2024 Mar 29;19(3):e0293858. doi: 10.1371/journal.pone.0293858 (PMC10980228; doi:10.1371/journal.pone.0293858)
Supplement: S1 File — (DOCX) [file pone.0293858.s001.docx]

**Supporting Information**

**Bicyclomycin generates ROS and blocks cell division in *Escherichia coli***

Anand Prakash^1,^* and Dipak Dutta^1^

^1^CSIR-Institute of Microbial Technology, Sector 39-A, Chandigarh 160036, India

Correspondence: [anan.prbt17@gmail.com](mailto:dutta@imtech.res.in)

**Supporting Figure S1: Triple mutant (Δ3) is more sensitive in presence of BCM.**

**
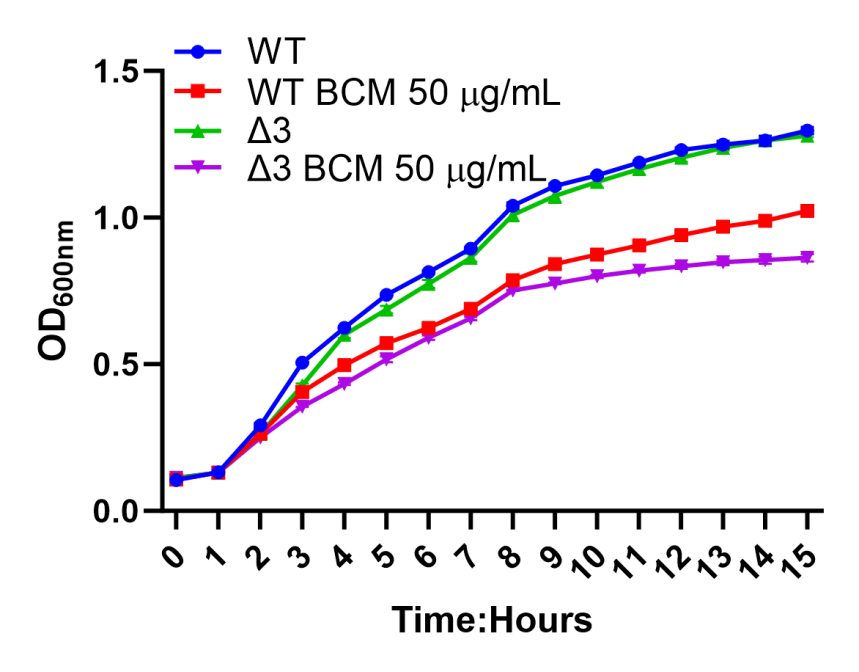
**

**Figure S1 : *ΔsodA ΔkatG ΔahpC* triple mutant (Δ3) is more sensitive to BCM.** WT and Δ3 strains displayed almost identical growth profiles in the absence of BCM. However, Δ3 strain showed more reduced growth in the presence of BCM compared to WT.

**Supporting Figure S2: Effect of tiron and thiourea on WT strain.**

**
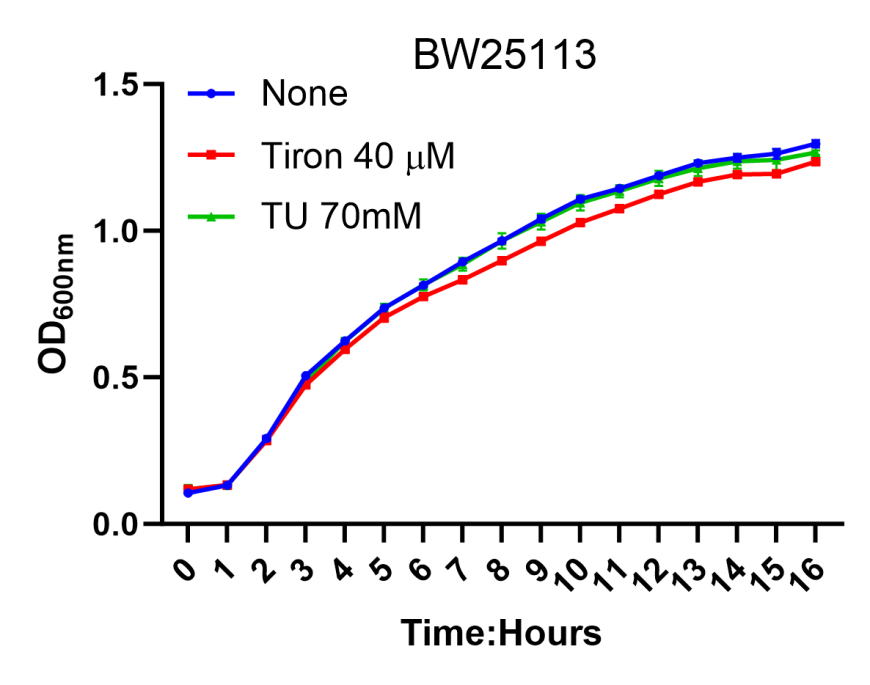
**

**Figure S2 : Checking effect of ROS scavengers, tiron and thiourea on the growth of WT BW25113.** WT cells grew almost identically without and with supplementation of thiourea. When tiron was added to the media, WT cells showed very minor slow growth.

**Supporting table 1. Expression level of genes involved in SOS response after BCM (µg/mL) treatment**

| Gene | 10 BCM F change | 50 BCM F change | 100 BCM F change | Description |
| --- | --- | --- | --- | --- |
| dinB | 3.37 | 3.94 | 7.96 | DNA polymerase IV |
| umuD | 4.10 | 5.58 | 5.68 | Subunit of DNA polymerase V |
| YdjQ (sosD) | 2.82 | 3.79 | 10.53 | Cho endonuclease, UvrC homologue |
| UvrD | 1.08 | 1.12 | 3.25 | DNA helicase II |
| ruvC | 2.10 | 1.80 | 1.51 | Holliday junction endonuclease |

**Supporting table 2. Expression level of genes involved in cell division after BCM treatment**

| Gene | 10 BCM F change | 50 BCM F change | 100 BCM F change | Description |
| --- | --- | --- | --- | --- |
| EzrA | 0.95 | 1.10 | 13.51 | Sensor histidine kinase ZraS |
| ftsB | 1.56 | 1.92 | 2.72 | Forms trimeric complex with FtsQ and FtsL |
| ftsX | 2.14 | 2.48 | 0.42 | Subunit of ABC transporter FtsEX, building divisome and peptidoglycan synthesis |
| envC | 2.66 | 2.42 | 0.73 | Cytoplasmic membrane protein binds FtsX |
| ftsH | 1.49 | 1.53 | 0.48 | Membrane bound protease |

**Supporting table 3. Expression level of genes involved in biofilm formation after BCM treatment**

| Gene | 10 BCM F change | 50 BCM F change | 100 BCM F change | Description |
| --- | --- | --- | --- | --- |
| luxS | 1.13 | 1.82 | 3.11 | synthesis of AI-2 |
| mqsA | 0.74 | 1.00 | 2.30 | Regulates biofilm formation |
| *yaiC* | 0.96 | 1.48 | 4.94 | cellulose biosynthesis |
| csgA | 2.42 | 3.06 | 10.74 | curli major subunit |
| *csgB* | 0.46 | 0.63 | 5.27 | curli minor subunit |
| *csgC* | 1.05 | 1.32 | 3.69 | curli production genes |
| *csgD* | 2.57 | 3.99 | 9.55 |  |
| *csgE* | 0.81 | 0.90 | 2.64 |  |
| *csgF* | 1.08 | 1.65 | 2.75 |  |
| *csgG* | 1.79 | 2.41 | 4.93 | curli production genes |
| sfmA | 9.35 | 21.78 | 47.31 | putative fimbrial protein |
| sfmH | 0.34 | 1.24 | 35.43 | putative fimbrial adhesin protein |
| ydeT | 3.87 | 8.10 | 99.46 | fimbrial usher domain-containing protein |
| fimZ | 2.95 | 4.05 | 9.20 | putative LuxR family transcriptional regulator |
| yfcV | 2.74 | 8.23 | 219.27 | putative fimbrial protein |
| elfA | 7.03 | 11.40 | 28.59 | putative laminin-binding fimbrial |
| *yadN* | 2.75 | 12.76 | 65.97 | putative fimbrial protein |
